# Ribociclib is not a substrate or inhibitor of Oatp1b-mediated uptake *in vivo*

**DOI:** 10.1007/s00280-026-04877-x

**Published:** 2026-04-07

**Authors:** Thomas Drabison, Eman A. Ahmed, Nathan Colasanti, Robert H. Weber, Alex Sparreboom, Eric D. Eisenmann

**Affiliations:** 1 Division of Pharmaceutics and Pharmacology, College of Pharmacy, The Ohio State University, Columbus, OH USA; 2 Comprehensive Cancer Center, The Ohio State University, Columbus, OH USA

**Keywords:** Ribociclib, DDI, OATP1B, CYP3A, Transport, Paclitaxel

## Abstract

**Purpose:**

Ribociclib is a CDK4/6 inhibitor used to treat HR+/HER2- breast cancer. Despite regulatory documents suggesting that ribociclib may inhibit both CYP3A and OATP1B-type transport *in vitro*, it is unclear whether CDK4/6 inhibitors interact with these mechanisms *in vivo*. Based on two cases of severe rhabdomyolysis in patients taking a CDK4/6 inhibitor and simvastatin, a CYP3A and OATP1B substrate, we tested the hypothesis that CDK4/6 inhibitors may precipitate drug-drug interactions through these mechanisms.

**Methods:**

We assessed the ability of CDK4/6 inhibitors to inhibit CYP3A and OATP1B-type transporters. Based on these data, we performed pharmacokinetic studies and toxicity assessments to determine whether ribociclib is a substrate or inhibitor of OATP1B-type transport *in vivo.*

**Results:**

Ribociclib inhibited the metabolism of triazolam, a CYP3A probe, *in vivo*. Additionally, CDK4/6 inhibitors inhibited OATP1B-type transporters *in vitro*. However, ribociclib, the most potent OATP1B inhibitor, did not influence the pharmacokinetics or pharmacodynamics of the OATP1B substrates CDCA-24G or paclitaxel. Furthermore, Oatp1b2 deficiency did not alter the pharmacokinetics of ribociclib.

**Conclusion:**

Our findings suggest that clinically significant OATP1B-mediated interactions are not anticipated with CDK4/6 inhibitors, either as victims or perpetrators, which supports ongoing clinical trials investigating the co-administration of CDK4/6 inhibitors with OATP1B substrates and inhibitors.

**Supplementary Information:**

The online version contains supplementary material available at 10.1007/s00280-026-04877-x.

## Introduction

Cyclin-dependent kinase 4/6 inhibitors (ribociclib, palbociclib, and abemaciclib) are components of the standard of care for the treatment of advanced hormone receptor-positive, human epidermal growth factor receptor 2-negative (HR+/HER2−) breast cancer [1, 2]. These agents are commonly co-administered with endocrine therapy (aromatase inhibitors [3–6], selective estrogen receptor degraders [7–9]), chemotherapy (platinum compounds [10, 11], taxanes [12]), targeted agents (tyrosine kinase inhibitors [13–17]), or immunotherapies [18–20].

Combining CDK4/6 inhibitors with other therapies increases the risk of drug-drug interactions (DDI) [21] through mechanisms including inhibition or induction of drug metabolic enzymes and transporters (DMETs). CDK4/6 inhibitors may be the victim or perpetrator of a DDI. For example, as a victim, the bioavailability of palbociclib is reduced by 62% when coadministered with a proton pump inhibitor [22, 23]. As a perpetrator, ribociclib inhibits MATE1/OCT2 transporters,causing metformin-induced acute kidney injury and lactic acidosis [24]. Moreover, CDK4/6 inhibitors are substrates and inhibitors of the cytochrome P450 enzyme CYP3A4, which has informed dosing recommendations in each of their package inserts. CDK4/6 inhibitors cause increased exposure of midazolam, a CYP3A4 substrate, suggesting inhibition of CYP3A4 [25]. Conversely, ritonavir, a prototypical inhibitor of CYP3A, increases CDK4/6 inhibitor exposure [26], albeit ritonavir also inhibits drug transporters that may be relevant to CDK4/6 inhibitor disposition [23].

Clinical case studies implicate CYP3A4 inhibition as the cause of severe rhabdomyolysis in patients receiving both CDK4/6 inhibitors and simvastatin [27, 28]. However, decreased hepatic transport of simvastatin may also contribute to this toxicity. Organic anion-transporting polypeptides 1B1 and 1B3 (OATP1B1 and OATP1B3) are key hepatic transporters that mediate the uptake of endogenous molecules and xenobiotics into hepatocytes [29]. Despite the potential for OATP1B inhibition to contribute to reported DDIs between CDK4/6 inhibitors and statins or other interactions with CDK4/6 inhibitors [30], interactions between CDK4/6 inhibitors and OATP1B transporters have not been comprehensively evaluated. This scientific gap provides the rationale for assessing OATP1B-mediated interactions as a contributor to CDK4/6 inhibitor-related toxicities [27, 28].

## Materials and methods

**Transport studies.** We used HEK293 cells overexpressing human OATP1B1, OATP1B3, mouse Oatp1b2, or an empty vector, and maintained them in Dulbecco’s Modified Eagle’s Medium (DMEM) supplemented with 10% FBS as previously described [31–33]. All cells were used within passage 30 and verified *Mycoplasma*-free using the MycoAlert Mycoplasma Detection Kit (Lonza). For inhibition assays, we plated 50,000 cells per well in a standard 96-well plate 24 h before the experiment. As previously described, we preincubated cells with CDK4/6 inhibitors at concentrations ranging from 6.25 to 100 µM for 15 min in warm, phenol red- and FBS-free DMEM. We then incubated the cells with the same concentrations of CDK4/6 inhibitors plus 8-FCA (8-(2-[fluoresceinyl]-aminoethylthio)-adenosine-3′,5′-cyclic-monophosphate), a prototypical OATP1B substrate [34], at a final concentration of 5 µM for an additional 15 min. Cells treated with vehicle and 8-FCA served as the baseline control, while untreated cells served as the assay blank. We terminated transport by washing the cells with ice-cold buffer. We quantified transport activity by spectrofluorometry (λ_ex_ = 485 nm, λ_em_ = 535 nm) and normalized the values to total protein using the Pierce BCA Protein Assay Kit (Thermo Fisher Scientific). We further standardized transport measurements to blank values and normalized them to the baseline uptake of 8-FCA.

Similarly, we utilized [^3^H]-pravastatin and [^3^H]-estradiol-17β-glucuronide (EβG) as a probes for OATP1B activity. Briefly, we plated 200,000 cells per well in standard 24-well plates 24 h before the experiment. We preincubated ribociclib at 1, 10, and 100 µM for 15 min in warm phenol red- and FBS-free DMEM. We then incubated the cells with the same concentrations of ribociclib plus [^3^H]-EβG a concentration of 0.2 µM for an additional 15 min as done previously [35] or [^3^H]-pravastatin at a concentration of 5 µM for an additional 10 min in accordance with experimentally determined parameters that fall in the linear range of pravastatin transport (**Supplementary Fig. 1**). Cells treated with vehicle and [^3^H]-probe served as baseline control. We terminated transport by washing cells with ice-cold PBS and lysed cells with 200 µL 1 M NaOH for at least 4 h at room temperature. Following lysis, lysates were neutralized with 100 µL 2 M HCl. To determine final radioactivity, 200 µL of neutralized lysate were transferred to scintillation vials containing 4 mL scintillation fluid, samples were mixed, and total radioactivity was determined by Tri-carb Liquid Scintillation Counter.

**Competitive counterflow assay.** A competitive counterflow (CCF) assay was employed using the radiolabeled probe EβG and OATP1B1-, Oatp1b2-, and OATP1B3-overexpressing cells according to a validated protocol [31]. Briefly, these cells were preloaded to saturation at room temperature with 0.01 µmol/L EβG in pre-warmed serum- and phenol red–free media for 1 h. Following loading, efflux of the radiolabeled probe resulted from the introduction of 1 µL stock solution of 0.1, 1, and 10 mmol/L test compound in DMSO. Following a 30-minute post-incubation of test compound, the transport activity was terminated by a 3× wash with 4 °C PBS. Finally, the cells were lysed with 200 µL 1 M NaOH for 4 h at room temperature. Following lysis, lysates were neutralized with 100 µL 2 M HCl. To determine final radioactivity, 200 µL of this solution were transferred to scintillation vials containing 4 mL scintillation fluid, samples were mixed, and total radioactivity was determined by Tri-carb Liquid Scintillation Counter.

**Animals.** We used 8–17-week-old, female, age-matched mice for this study. Animals were housed in standard, temperature-controlled cages with a 12-hour light/dark cycle, and had access to a standard diet and water *ad libitum*. We followed housing and handling guidelines set by the University Laboratory Animal Resources Animal Care and Use Committee at The Ohio State University (OSU), under approved animal protocols (2015A00000101-R2 and 2024A00000067). Wild-type FVB mice were purchased from Taconic, and Oatp1a/1b(-/-) mice were provided by Dr. Alfred Schinkel [36].

**Pharmacokinetic studies.** We performed pharmacokinetic studies using an established protocol [37]. We fasted mice for 3 h before administering oral drugs. We orally administered either 50 or 100 mg/kg ribociclib succinate hydrate or vehicle (0.9% saline). We administered paclitaxel or vehicle [50% Kolliphor EL (polyoxyethylene glycerol triricinoleate 35; formerly known as Cremophor EL) and 50% ethanol, diluted 1:2 in 0.9% saline] as a single intravenous dose at 10 mg/kg. We also administered triazolam (formulated in 1:99 ethanol and 0.9% saline) as a single 2 mg/kg oral dose. Finally, we administered pravastatin (formulated in 0.9% saline) as a single 20 mg/kg oral dose. We dosed all drugs at 5 µL/g body weight. To capture the maximal plasma concentration (C_max_) and terminal phase for each route, we collected serial blood samples based on historical pharmacokinetic data [38]: ribociclib (0.25, 0.5, 1, 3, 6, and 12 h), paclitaxel (3, 7, 15, 30, 60, and 120 min), triazolam (5, 10, 20, 40, 80, and 160 min), and pravastatin (2, 5, 15, 30, and 60 min). We collected whole blood samples from the submandibular vein for the first three blood collections and from the retro-orbital sinus vein for the last three. After centrifuging the blood samples at 13,000 × *g* for 5 min, we collected the plasma supernatants and stored them at -80 °C until analysis. At the endpoint, we collected dorsal root ganglia (DRG) and liver tissues, then snap-froze them using dry ice to prevent ongoing metabolic activity. We homogenized DRG and liver tissues with 5-mm stainless steel beads (Qiagen, Hilden, Germany) for 4 min at 40 Hz. DRG tissues were weighed and homogenized in 20 µL LC-MS grade water; liver tissues were weighed and homogenized in 1:3 (mass: volume) LC-MS grade water. After removing the beads, we centrifuged the homogenized samples at 13,000 × *g* for 5 min and stored the supernatants at − 80 °C until analysis.

**Quantification of ribociclib using LC-MS/MS.** Ribociclib (purity 99.99%, Selleckchem, TX, USA) and its internal standard [^2^H_6_]-ribociclib (purity > 98%, Alsachim, France) were quantified using a Vanquish Ultra-High-Performance Liquid Chromatography (UHPLC) system coupled with an TSQ Altis™ Triple Quadrupole Mass Spectrometer (Thermo Fisher Scientific, USA). Chromatographic separation was achieved on an Acquity UPLC HSS T3 Column (100Å, 1.8 μm, 2.1 mm X 50 mm, Waters Co.) equipped with an Acquity UPLC HSS T3 Vanguard pre-column (1.8 μm, 2.1 mm X 5 mm, Waters Co.). The column was maintained at 40 °C and the autosampler at 4 °C. The mobile phase consisted of solvent A (0.1% formic acid in LC-MS grade water) and solvent B (0.1% formic acid in LC-MS grade acetonitrile), with a total run time of 4.5 min and a flow rate of 0.4 mL/min. A diverter valve was used to direct flow into the mass spectrometer only between 0.5 and 3.0 min to reduce impurity interference. Mass spectrometric detection was performed using heated electrospray ionization (ESI) in positive ionization mode, with selective reaction monitoring (SRM). The mass spectrometer was operated under the following conditions: the auxiliary gas was set at 10 arbitrary units (Arb), sheath gas at 50 Arb, and sweep gas at 1 Arb. Argon was used as the collision gas at a pressure of 1.5 mTorr. The vaporizer temperature was maintained at 380 °C, while the ion transfer tube temperature was set at 350 °C. A positive ion spray voltage of 1500 V was applied.

The method was optimized using a 0.5 µg/mL standard mixture of ribociclib and [^2^H_6_]-ribociclib in acetonitrile, and SRM transitions were set at m/z 435.24→ 321.97 for ribociclib and m/z 441→ 321.97 for the internal standard. Data acquisition and processing were performed using Thermo Scientific Xcalibur software (version 4.4.16.14). An eight-point non-zero calibration curve (5–1000 ng/mL) was constructed and analyzed over four consecutive days, with two calibration runs daily. Calibration curves were fitted using a 1/x² weighted linear regression model, achieving excellent linearity (r² > 0.99). Quality control (QC) samples were prepared at five concentration levels: LLOQ (5 ng/mL), LQC (15 ng/mL), MQC (150 and 450 ng/mL), HQC (850 ng/mL), and AULQ (8500 ng/mL), with AULQ samples diluted tenfold in blank mouse plasma prior to analysis. The method was fully validated and complied with the FDA bioanalytical method validation guidelines [39]. The method showed acceptable accuracy, with bias (%) values of 6.5% at LLOQ and ranging from − 1.7% to 4.1% across the other four QC levels.

Sample preparation was performed using a protein precipitation technique, where 10 µL of mouse plasma was mixed with 5 µL of internal standard solution (0.1 µg/mL) and 85 µL of methanol, vortexed for 30 s, and centrifuged at 13,000 × *g* for 10 min at 4 °C. A 70 µL aliquot of the supernatant was transferred to a WebSeal™ 96-well plate (Thermo Scientific, MA, USA), sealed, and 5 µL was injected for analysis.

**Quantification of paclitaxel**,** CDCA-24G**,** triazolam**,** OH-triazolam**,** and pravastatin using LC-MS/MS.** Concentrations of paclitaxel, CDCA-24G, triazolam, α-hydroxytriazolam (OH-triazolam) and pravastatin in mouse plasma were measured using previously validated LC-MS/MS methods [40–43].

**Pharmacokinetic data analysis.** We performed non-compartmental analysis to determine the pharmacokinetic parameters for paclitaxel using Lixoft PKanalix (Antony, France, 2024R1). We determined C_max_ values by visually inspecting the log concentration versus time curves. We used the linear trapezoidal rule to calculate the area under the plasma concentration-time curve (AUC) from time zero to the last measurable concentration (AUC₀_–last_) or extrapolated to infinity (AUC_∞_).

**Peripheral neuropathy assessment.** Mice were administered a single intravenous dose of paclitaxel (10 mg/kg) or vehicle. We assessed nocifensive behavior in response to mechanical stimulation using the von Frey Hairs (VFH) test to evaluate mechanical allodynia at 6 and 24 h after drug administration. We housed the mice individually in elevated cages with clear access to their hind paws and acclimated them to the VFH testing apparatus one day before the experiment. We recorded baseline measurements before dosing. We conducted behavioral testing in a quiet room following previously described procedures [44, 45].

Before testing, we placed the mice in elevated enclosures and allowed them to acclimate for 1 h. We applied a filament connected to an electronic esthesiometer (MouseMet, Topcat Metrology) perpendicularly to the plantar surface of the hind paw with a constant force. When the mice spontaneously withdrew their hind paws, we stopped the stimulus and recorded the force (g) required to elicit withdrawal. We performed this procedure three times by applying the filament to the mid-plantar region of each hind paw from beneath the mesh floor, with a few seconds between trials. We used individual baseline values for each mouse and set an upper cutoff limit of 6 g to prevent tissue damage.

**Data analysis.** All data are expressed as the mean ± standard deviation (SD), normalized to baseline values. We used an unpaired two-sided Student’s t-test with Welch’s correction to analyze normally distributed data and applied a Kolmogorov-Smirnov test for non-normally distributed data when comparing two groups. Normality was tested using the Shapiro-Wilk Test. For comparisons involving more than two groups, we used an ordinary one-way ANOVA. We analyzed behavioral data using two-way and three-way ANOVA with Bonferroni’s *post hoc* test, repeated across time points and groups. We considered *P* < 0.05 statistically significant for all analyses.

## Results

**Concentration-dependent inhibition of OATP1B-mediated transport by CDK4/6 inhibitors** ***in vitro*.** We evaluated whether CDK4/6 inhibitors inhibit OATP1B-mediated transport by exposing overexpressing cell lines (OATP1B1, Oatp1b2, OATP1B3) and empty vector controls to increasing concentrations of ribociclib, palbociclib, and abemaciclib for 15 min, then co-incubating the CDK4/6 inhibitors with 8-FCA. CDK4/6 inhibitors inhibited 8-FCA uptake in a concentration-dependent manner (Table 1, **Supplementary Fig. 2**). To determine the clinical relevance of IC_50_ values for ribociclib (25.9, 19.2, & 42.9 µM), abemaciclib (24.9, 25.7, & 41.1 µM), and palbociclib (46.1, 55.2, & 77.5 µM), we calculated R values for OATP1B1 and 1B3 for each CDK4/6 inhibitor as suggested by the FDA [46]. We utilized clinical C_max, ss_ values determined previously (**Supplementary Table 1**). In accordance with FDA guidance, we converted the dose or C_max, ss_ to µM and determined the unbound fraction in plasma. Additionally, we set the fraction absorbed and intestinal availability to 1 and the absorption constant to 0.1 min^− 1^ to represent the worst-case scenario. Hepatic blood flow rate was set to 1.62 L/min. R values greater than 1.1 indicate that the drug has *in vivo* potential to inhibit the transporter. Based on an R value > 1.1 for ribociclib with both OATP1B1 and OATP1B3 (Table 1), we selected ribociclib for further *in vivo* analysis.

**Table 1:** IC_50_ Values for CDK4/6 inhibitors and OATP1B Transporters

**Inhibitor**	OATP1B1		Oatp1b2		OATP1B3	
	IC_50_ (µM) (95% CI)	*R* Value	IC_50_ (µM) (95% CI)	*R* Value	IC_50_ (µM) (95% CI)	*R* Value
**Ribociclib**	25.9 (21.4–30.4)	2.00	19.2 (15–23.4)	NA	42.9 (43.9–54.5)	1.61
**Abemaciclib**	24.9 (19.5–30.3)	1.06	25.7 (18.9–32.5)	NA	41.1 (29.9–52.3)	1.04
**Palbociclib**	46.1 (21.6–70.7)	1.06	55.2 (50.6–59.8)	NA	29.5 (28.6–30.4)	1.03

R values calculated as described in FDA guidance [46]

As a secondary validation and to rule out substrate-specific interactions, we verified this result utilizing [^3^H]-EβG and [^3^H]-pravastatin, radiolabeled probes of OATP1B-transport function [47]. After a 15-minute preincubation with increasing concentrations of ribociclib, 0.2 µM [^3^H]-EβG was coincubated at the same concentrations of ribociclib for 15 min [35]. Similarly, 5 µM [^3^H]-pravastatin was co-incubated for 10 min before the experiment was terminated. These limited concentration gradients of ribociclib verify concentration-dependent inhibition of OATP1B-mediated transport *in vitro* (**Supplementary Fig. 3**).

**Determining the dose of ribociclib** ***in vivo***. To assess the impact of ribociclib on CYP3A-mediated metabolism and OATP1B-mediated transport, we conducted a preliminary pharmacokinetic analysis to determine an optimal dose and schedule for studying potential interactions. Consistent with previous studies [48], we administered a single oral dose of ribociclib at 50 or 100 mg/kg to wild-type mice. Then, plasma and liver samples were analyzed for ribociclib concentrations via LC-MS/MS (**Supplementary Fig. 4**,** Supplementary Table 2**). We found that ribociclib exhibits dose-linear pharmacokinetics between 50 mg/kg and 100 mg/kg, with a fold change in C_max_ of 1.6 (50 vs. 100 mg/kg; 4.7 ± 1.2 vs. 8.0 ± 3.2 µg/mL) and AUC₀_–last_ of 2.3 (50 vs. 100 mg/kg; 21 ± 11 vs. 49 ± 28 µg*h/mL). Liver accumulation is also dose-dependent (50 vs. 100 mg/kg; 14 ± 3.4 vs. 33 ± 19 µg/g) (**Supplementary Fig. 4c)**. At a C_max_ of 8.0 µg/mL (18.4 µM), 100 mg/kg ribociclib surpassed clinically relevant concentrations [49], representing a worst-case scenario and was chosen for all subsequent studies.

**Fig. 1:**
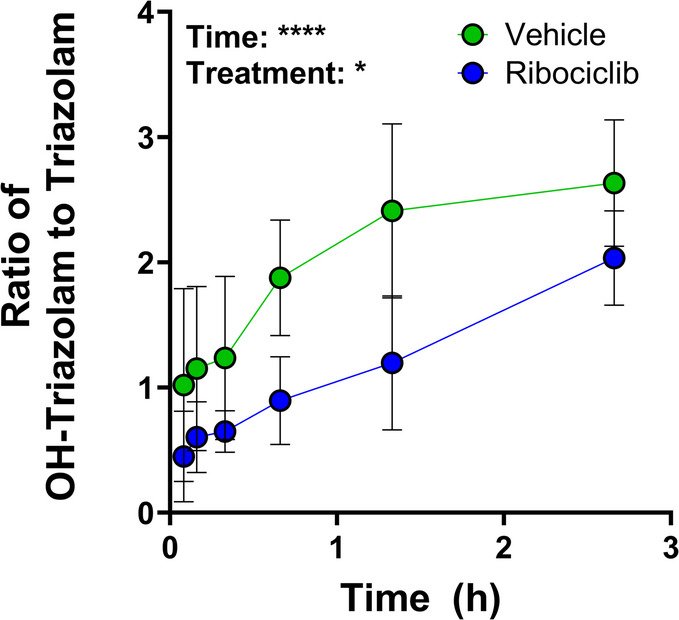
Inhibition of CYP3A by ribociclib. OH-triazolam: triazolam ratio in mice treated with ribociclib (100 mg/kg; *p.o.*) or vehicle. *n* = 5 per group, error bars represent SD

**Ribociclib decreases CYP3A mediated metabolism.** To validate whether CDK4/6 inhibitors inhibit CYP3A, we treated mice with ribociclib (100 mg/kg, *p.o*.) or vehicle, followed 30 min later by an oral dose of triazolam (2 mg/kg), a validated probe for CYP3A enzymatic activity in mice [50]. Serial blood samples were collected and analyzed by LC-MS/MS to quantify both parent triazolam and its CYP3A-dependent metabolite, OH-triazolam [51]. As expected, ribociclib reduced CYP3A-mediated conversion of triazolam to OH-triazolam, as indicated by a significant difference in the metabolite-to-parent ratio in mice treated with ribociclib compared to vehicle (Fig. 1, **Supplementary Table 3**). The metabolite-to-parent ratio of triazolam to OH-triazolam was significantly impacted by both time (*P* < 0.0001) and treatment (ribociclib or vehicle) (*P* < 0.05) as determined by mixed-effects analysis.

The observed clinical DDI between CDK4/6 inhibitors and simvastatin [27, 28] may involve factors in addition to CYP3A inhibition, especially considering the relatively modest effect on CYP3A metabolism observed in our studies. Given the observed *in vitro* inhibition by CDK4/6 inhibitors and the known role of hepatic transporters in the disposition of statins [52], we next examined whether ribociclib affects OATP1B-mediated transport *in vivo*.

**Fig. 2:**
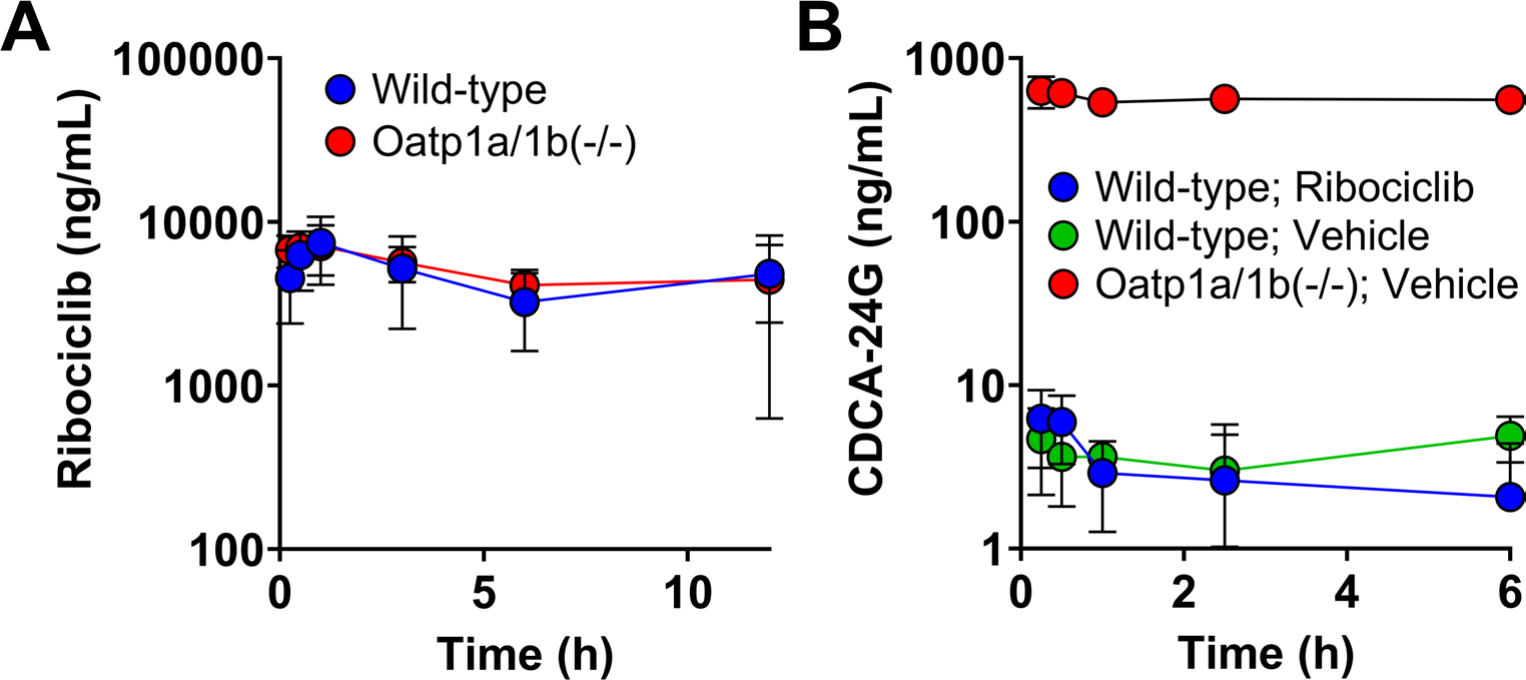
*In vivo* evaluation of ribociclib as a substrate or inhibitor of OATP-type transporters. **(A)** Plasma concentration-time profiles of 100 mg/kg ribociclib (*p.o.*) in wild-type or Oatp1a/1b(-/-) mice. **(B)** Plasma concentration-time profile of CDCA-24G in wild-type or Oatp1a/1b(-/-) mice in the presence of 100 mg/kg ribociclib or its vehicle. *n*=5 per group, errors bars represent SD

**Ribociclib does not significantly interact with OATP1B** ***in vivo***. To evaluate whether ribociclib is an OATP1B substrate, we compared the pharmacokinetic profile of wild-type mice and mice lacking OATP1A- and OATP1B-type transporters (Oatp1a/1b(-/-)). Ribociclib (100 mg/kg, *p.o*.) was administered to both groups, and plasma concentrations were quantified via LC-MS/MS. Ribociclib pharmacokinetics were unchanged between wild-type and Oatp1a/1b(-/-) mice, with comparable C_max_, AUC, and liver accumulation values (wild-type vs. Oatp1a/1b(-/-); C_max_: 8.0 ± 3.2 vs. 7.8 ± 1.4 µg/mL; AUC_0-__last_ : 49 ± 28 vs. 49 ± 15 µg*h/mL; liver accumulation: 33 vs. 44 µg/g) (*P* > 0.05) (Fig. 2a, **Supplementary Table 2**,** Supplementary Fig. 4**).

As a secondary validation of this result, we utilized an established competitive counterflow assay [31]. We saturated cells that overexpress OATP1B1, Oatp1b2, and OATP1B3 with the radiolabeled probe [3 H]-EβG. Once saturation is achieved, introduction of ribociclib did not induce an efflux of the loaded probe, compared to unlabeled EβG as a positive control (**Supplementary Fig. 5**), validating the *in vivo* result, indicating that ribociclib is not a transported substrate of OATP1B-type transporters.

To determine whether ribociclib inhibits OATP1B transport *in vivo*, we measured plasma levels of CDCA-24G, an endogenous OATP1B biomarker [53], after ribociclib administration. We administered either ribociclib or vehicle to wild-type and Oatp1a/1b(-/-) mice and analyzed serial blood samples via LC-MS/MS. We did not observe a significant difference in CDCA-24G concentrations between ribociclib- and vehicle-treated wild-type mice (vehicle- vs. ribociclib-treated; C_max_: 6.2 ± 1.8 vs. 7.6 ± 1.4 ng/mL; AUC_last_ : 21.6 ± 7.1 vs. 10.6 ±10.9 ng*h/mL) (*P*>0.05). As expected, CDCA-24G levels were higher in Oatp1a/1b(-/-) mice regardless of treatment (wild-type vs. Oatp1a/1b(-/-); C_max_: 6.2 ± 1.7 vs. 700 ± 50 ng/mL; AUC_0-__last_ : 21.6 ± 7.1 vs. 4,400 ± 3,600 ng*h/mL) (Fig. 2b, **Supplementary Table 4**). Additionally, to verify that ribociclib does not inhibit OATP1B-type transporters and to rule out substrate-specific interactions, we utilized pravastatin as a clinically relevant exogenous probe of OATP1B transport activity. As before, we administered either ribociclib or vehicle to wild-type and Oatp1a/1b(-/-) mice prior to pravastatin administration (20 mg/kg; *p.o.*) and analyzed serial blood samples via LC-MS/MS. We did not observe a significant difference in pravastatin concentrations between ribociclib- and vehicle-treated wild-type mice (vehicle- vs. ribociclib-treated; C_max_: 69 ± 52 vs. 37 ± 32ng/mL; AUC_last_: 45 ± 19 vs. 22 ± 7 ng*h/mL) (*P*>0.05). As expected, pravastatin levels were higher in Oatp1a/1b(-/-) mice compared to wild-type (wild-type vs. Oatp1a/1b(-/-)); C_max_: 69 ± 52 vs. 4669 ± 1480 ng/mL; AUC_last_: 45 ± 19 vs. 3606 ± 846 ng*h/mL) (**Supplementary Fig. 6**,** Supplementary Table 5**) 

**Ribociclib does not induce pharmacokinetic or pharmacodynamic DDI with paclitaxel.** Paclitaxel is a cytotoxic chemotherapeutic used as front-line therapy in breast cancer [55], and is being evaluated in combination with CDK4/6 inhibitors [30, 56]. Paclitaxel causes a dose-dependent peripheral neuropathy that is sensitive to changes in OATP1B, despite minimal changes in paclitaxel plasma concentrations with OATP1B inhibition [32]. To evaluate potential pharmacodynamic DDIs mediated by OATP1B transporters, we assessed paclitaxel-induced peripheral neuropathy in the presence of ribociclib or its vehicle. Mice received 100 mg/kg ribociclib (*p.o.*) or vehicle 30 min before a single 10 mg/kg IV bolus of paclitaxel or its vehicle in either wild-type or Oatp1a/1b(-/-) mice.

**Fig. 3:**
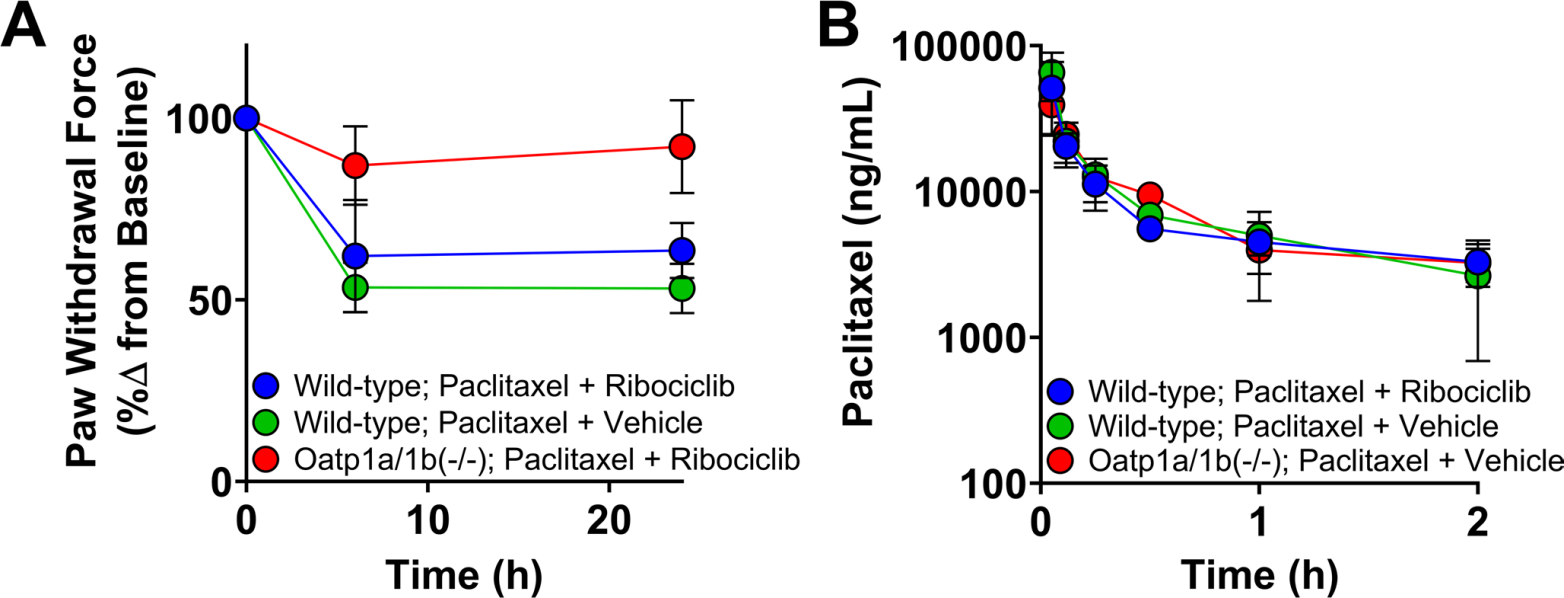
DDI potential of ribociclib through type transporters. **(A)** VFH and **(B)** plasma concentration time profiles of paclitaxel in wild-type or Oatp1a/1b(-/-) mice in the presence of ribociclib or its vehicle. *n* = 4–6 per group, error bars represent SD

Neither ribociclib nor its vehicle altered paclitaxel-induced peripheral neuropathy (Fig. 3a). Paclitaxel pharmacokinetics were unchanged between ribociclib- and vehicle-treated groups (vehicle- vs. ribociclib-treated; C_max_: 65 ± 24 vs. 51 ± 26 µg/mL; AUC_last_: 18 ± 3.7 vs. 17 ± 4.8 µg*h/mL) (*P* > 0.05) (Fig. 3b, **Supplementary Table 6**). In addition, ribociclib did not alter paclitaxel concentrations at the dorsal root ganglion (DRG), a site of injury for neuropathy, or liver in wild-type mice (vehicle- vs. ribociclib-treated; DRG: 109 ± 21 vs. 124 ± 30 ng/g; Liver: 40,000 ± 4,600 vs. 38,000 ± 6,900 ng/g) (*P* > 0.05) (**Supplementary Fig. 7**).

In contrast, and as expected, Oatp1a/1b(-/-) mice were protected from paclitaxel-induced neuropathy without a significant change in plasma pharmacokinetics (wild-type vs. Oatp1a/1b(-/-); C_max_: 65 ± 24 vs. 39 ± 15 µg/mL; AUC_last_: 18 ± 3.7 vs. 15 ± 6.0 µg*h/mL) (Fig. 3, **Supplementary Table 6**). This is consistent with previous transporter-mediated protection against neuropathy [32]. As expected, neuropathy did not develop in control groups that received the vehicle for paclitaxel or in Oatp1a/1b-deficient mice (**Supplementary Fig. 7c**).

## Discussion

Evaluating potential DDIs is critical for patient safety, particularly in combination therapies [58]. While *in vitro* DDI assessments are performed according to FDA requirements [46], comprehensive *in vivo* evaluations are rarely performed before combinations reach the market. Ribociclib (Kisqali) inhibits CYP3A and OATP1B1/3 *in vitro* [59, 60]. Our study confirms that ribociclib significantly reduces the CYP3A-mediated metabolism of triazolam, consistent with its known role as a CYP3A inhibitor [61]. However, based on clinical reports of CDK4/6 inhibitor-statin interactions [27, 28], concurrent OATP1B inhibition could contribute to CDK4/6 inhibitor-induced DDIs. To address this, we comprehensively evaluated the interaction potential between CDK4/6 inhibitors and OATP1B transporters.

Here, we demonstrate that CDK4/6 inhibitors inhibit OATP1B1/2/3 *in vitro*, and ribociclib’s R values suggest potential *in vivo* relevance. However, our follow-up experiments provide the first rigorous *in vivo* evidence that ribociclib does not cause clinically meaningful OATP1B inhibition. We chose a relatively high dose of ribociclib to simulate clinical scenarios where ribociclib exposure is elevated [62, 61] and to evaluate a worst-case scenario for a DDI. Despite this, we observed no significant changes in transporter activity.

Beyond pharmacokinetics, we also assessed the potential for OATP1B-mediated pharmacodynamic interactions. Given our expertise in studying paclitaxel-induced peripheral neuropathy [40] and evidence that paclitaxel is an OATP1B substrate [32,63], we leveraged this model to assess the potential of ribociclib as an OATP1B-mediated DDI perpetrator. Paclitaxel-induced peripheral neuropathy correlates with drug accumulation in dorsal root ganglia, rather than plasma levels, making it a sensitive marker for transporter disruption [40]. Ribociclib did not protect against paclitaxel-induced peripheral neuropathy, and its pharmacodynamic profile did not phenocopy that of Oatp1a/1b(-/-) mice, which are fully protected despite no plasma pharmacokinetic change. Additionally, ribociclib did not influence the Oatp1b-mediated hepatic transport of either CDCA-24G or paclitaxel.

Despite our findings, this study cannot rule out that altered transport mechanisms, independent of CDK4/6 inhibitor inhibition, contribute to the reported case studies. Within these case reports, comprehensive genetic analysis is unavailable for these patients [27, 28]. Given that genetic polymorphisms in OATP1B1 are well-documented risk factors for statin-induced myopathy, their potential contribution in these cases cannot be excluded. Single-nucleotide polymorphisms (SNPs), such as rs4363657C and rs4149056C and haplotypes SLCO1B1*15 and SLCO1B1*5 have been associated with reduced OATP1B1 transport activity, leading to increased systemic statin exposure and heightened risk of myopathy and rhabdomyolysis [64–67]. Future clinical studies should incorporate comprehensive pharmacogenomic analysis of key drug-metabolizing enzymes and transporters (DMETs) to better delineate the genetic contributions to altered statin pharmacokinetics and toxicity.

Our study provides strong evidence that ribociclib does not significantly inhibit OATP1B transporters *in vivo*, reinforcing its safety when coadministered with OATP1B substrates. However, its CYP3A inhibition highlights the need for caution when administered with CYP3A-metabolized drugs. Future investigations should explore patient-specific risk factors, such as genetic polymorphisms in CYP3A or OATP1B transporters, to further refine dosing recommendations and mitigate potential adverse effects in clinical practice. Nonetheless, these results clarify the role of CDK4/6 inhibitors in transporter-mediated interactions, facilitating safer combination therapies in oncology.

## Supplementary Information

Below is the link to the electronic supplementary material.

Supplementary Material 1

## Data Availability

The data supporting the findings of this study are available in the Dryad digital repository at https://doi.org/10.5061/dryad.f1vhhmh9d.
