# Supplementary material for: Ribociclib is not a substrate or inhibitor of Oatp1b-mediated uptake in vivo
Source: Cancer Chemother Pharmacol. 2026 Apr 7;96(1):32. doi: 10.1007/s00280-026-04877-x (PMC13056735; doi:10.1007/s00280-026-04877-x)
Supplement: Supplementary file 1 — Supplementary Material 1 [file 280_2026_4877_MOESM1_ESM.docx]

**Supplementary Material – Cancer Chemotherapy and Pharmacology**

**Ribociclib is not a substrate or inhibitor of Oatp1b-mediated uptake *in vivo***

Thomas Drabison^1^, Eman A. Ahmed^1^, Nathan Colasanti^1^, Robert H. Weber^1^, Alex Sparreboom^1,2^, and Eric D. Eisenmann^1,2,#^

**Authors’ Affiliations:**

^1^Division of Pharmaceutics and Pharmacology, College of Pharmacy, The Ohio State University, Columbus, OH, USA

^2^Comprehensive Cancer Center, The Ohio State University, Columbus, OH, USA

**Running title**: Ribociclib and Oatp1b

**Keywords:** Ribociclib, DDI, OATP1B, CYP3A, Transport, Paclitaxel

**Corresponding Author:** ^#^Eric D. Eisenmann, Division of Pharmaceutics and Pharmacology, College of Pharmacy & Comprehensive Cancer Center, The Ohio State University, Columbus, OH 43210; Phone: 440-991-6332; Email: [Eisenmann.11@osu.edu](mailto:Eisenmann.11@osu.edu)

**
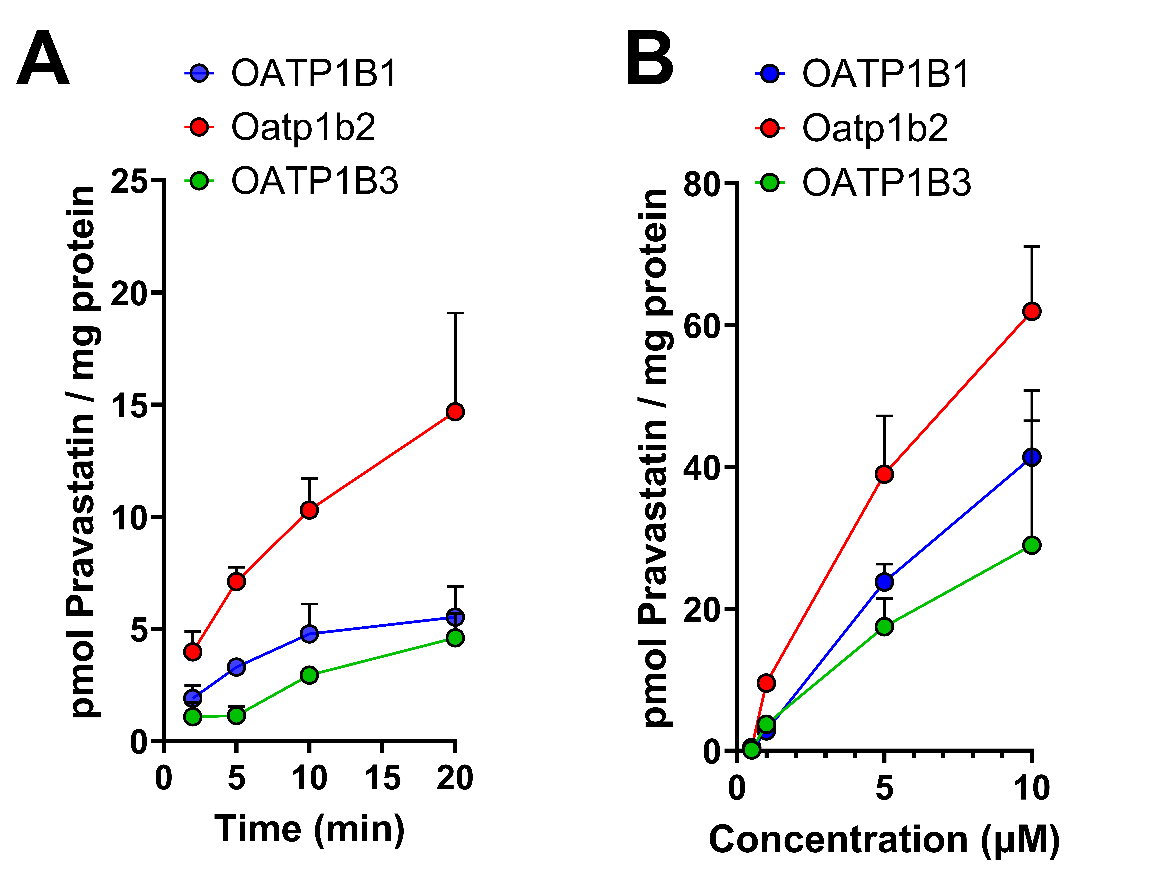
**

**Supplementary Fig. 1** (**A**) Time- and (**B**) concentration-dependent uptake of pravastatin in OATP1B1, Oatp1b2, and OATP1B3 cells determined by liquid scintillation counting to determine uptake parameters. n=3 across 1 biological replicates, error bars represent SD.

**
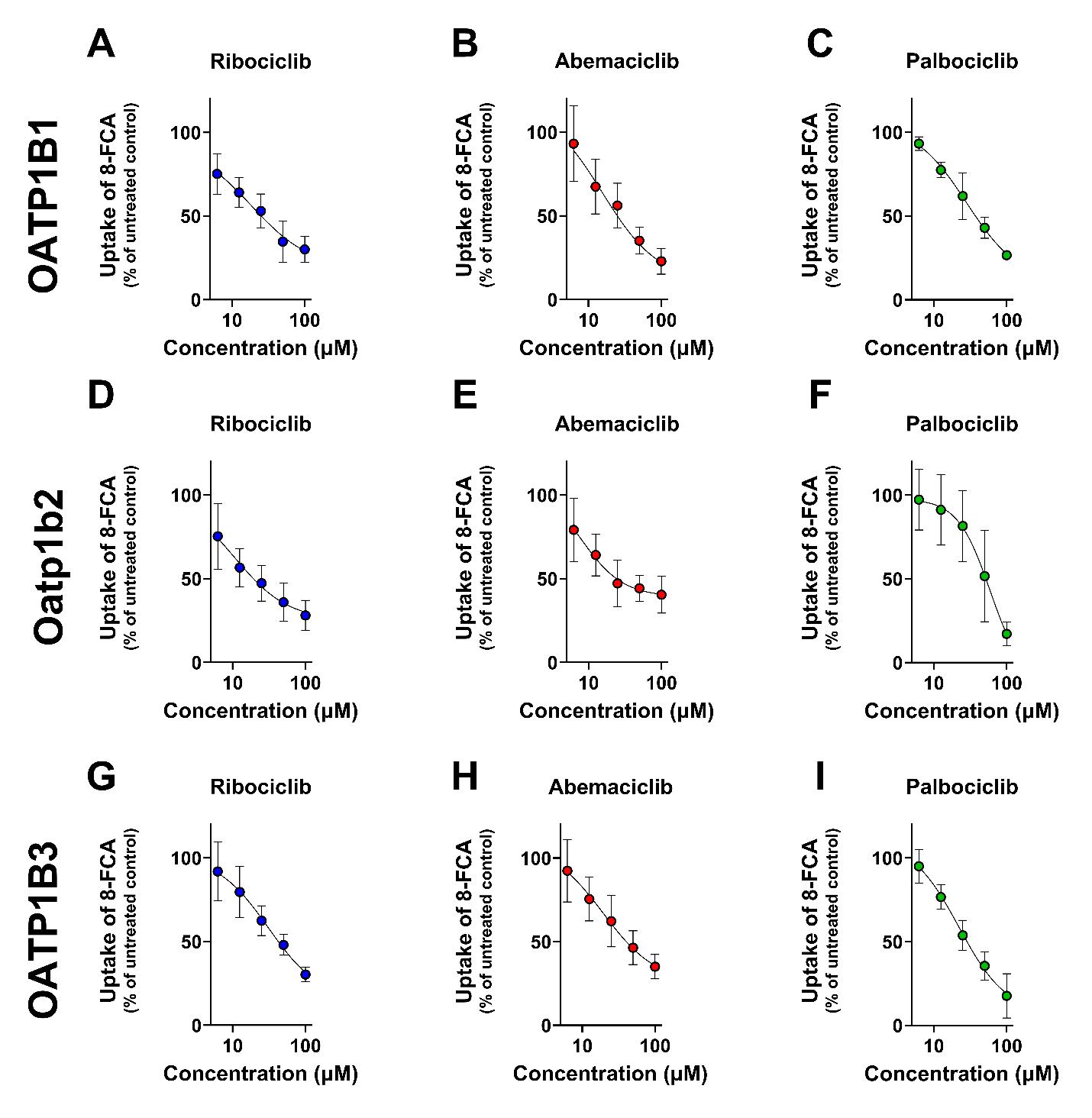
**

**Supplementary Fig. 2** Inhibition of OATP1B1, Oatp1b2, and OATP1B3 by ribociclib, palbociclib, and abemaciclib. Concentration curves for (**A**, **B**, & **C**) OATP1B1, (**D**, **E**, & **F**) Oatp1b2, and (**G**, **H**, & **I**) OATP1B3 with (**A**, **D**, & **G**) ribociclib, (**B**, **E**, & **H**) palbociclib, and (**C**, **F**, & **I**) abemaciclib. n=8-12 technical replicates across 2 biological replicates, error bars represent SD. Dose–response curves were fit using a four-parameter logistic (4PL) sigmoidal model with concentration as the independent variable (X-axis), using nonlinear least-squares regression.


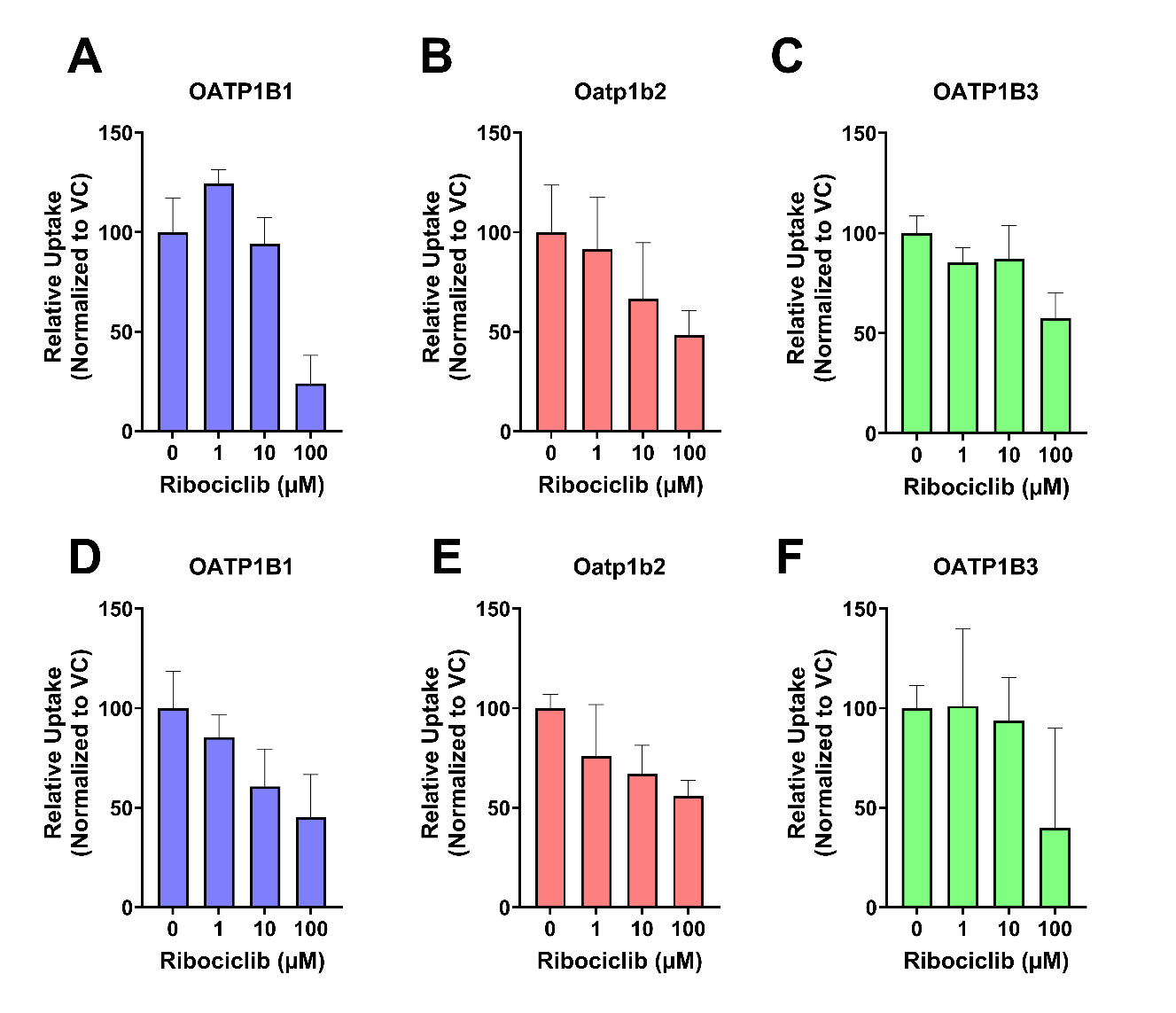


**Supplementary Fig. 3** Inhibition of (**A & D**) OATP1B1, (**B & E**) Oatp1b2, and (**C & F**) OATP1B3 by ribociclib, utilizing (**A, B, & C**) radiolabeled estradiol-17β-glucuronide (EβG) and (**D, E, & F**) pravastatin as probes. n=6 technical replicates across 2 biological replicates, error bars represent SD.


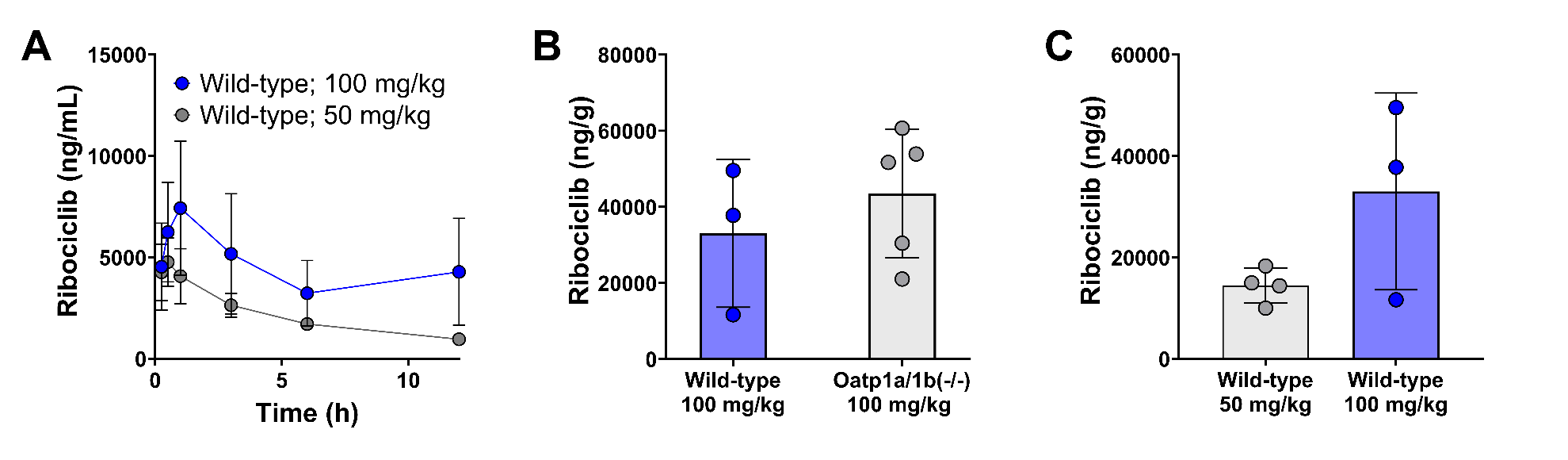


**Supplementary Fig. 4** Pharmacokinetics of ribociclib in wild-type and Oatp1a/1b(-/-) mice. (**A**) Concentration-time profile of 50 or 100 mg/kg ribociclib administered to wild-type mice (n=5/group). (**B**) Liver accumulation of 100 mg/kg ribociclib administered to wild-type or Oatp1a/1b(-/-) mice. (**C**) Liver accumulation of 50 or 100 mg/kg ribociclib administered to wild-type mice. Error bars represent SD.


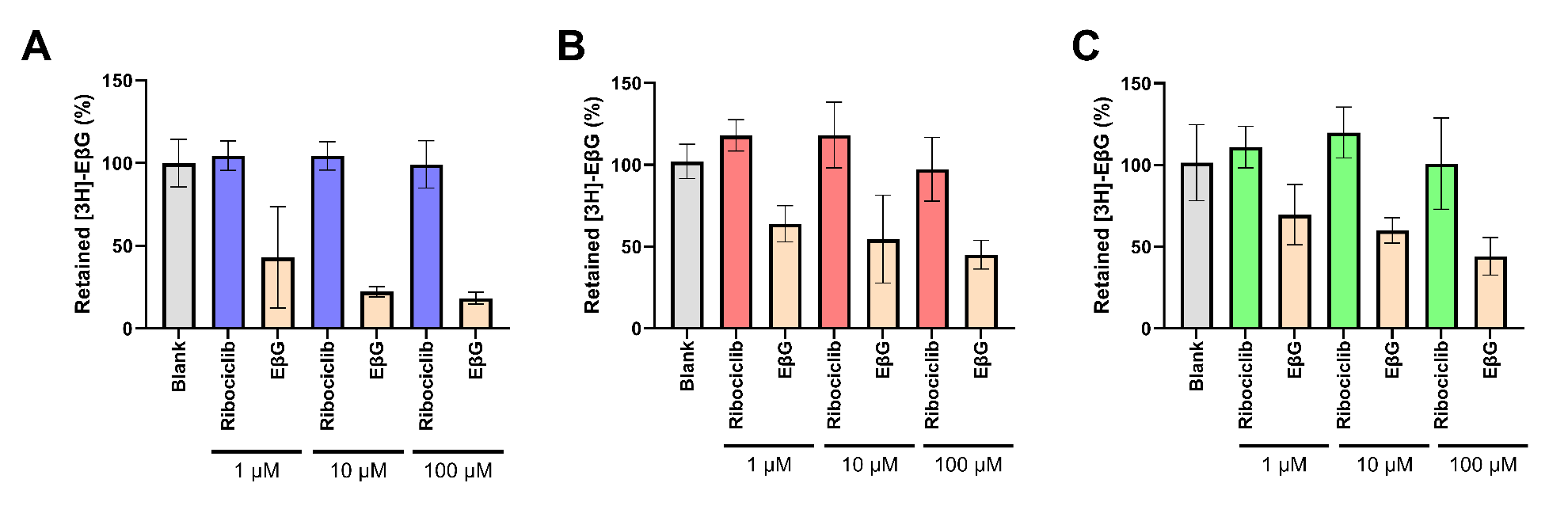


**Supplementary Fig. 5** Stimulated efflux of preloaded [^3^H]-EβG after the addition of 1, 10, or 100 μM ribociclib to (**A**) OATP1B1, (**B**) Oatp1b2, and (**C**) OATP1B3 cells. Final intracellular radioactivity was measured and presented relative to the efflux induced by an equimolar concentration of EβG, a known substrate and efflux inducer. n=6 technical replicates across n=2 biological replicates, error bars represent SD.


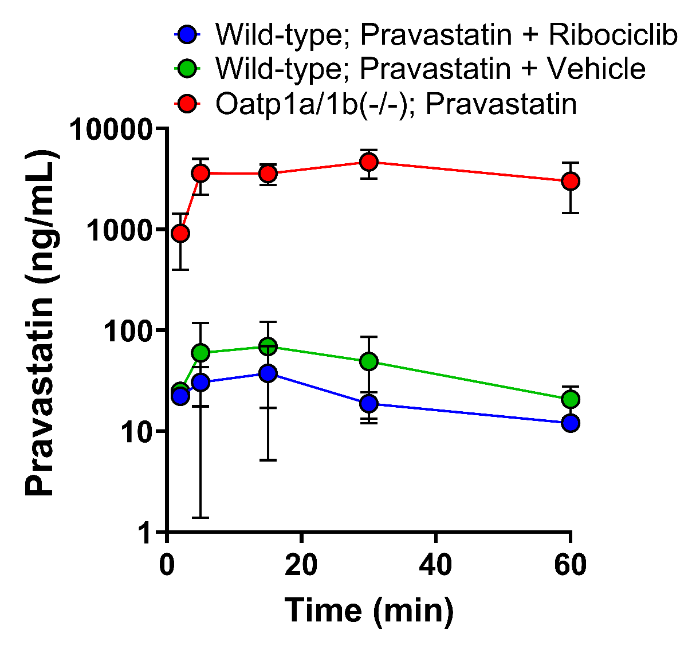


**Supplementary Fig. 6** Pharmacokinetics of pravastatin in wild-type and Oatp1a/1b(-/-) mice in the presence of ribociclib or its vehicle. n=5/group, error bars represent SD.


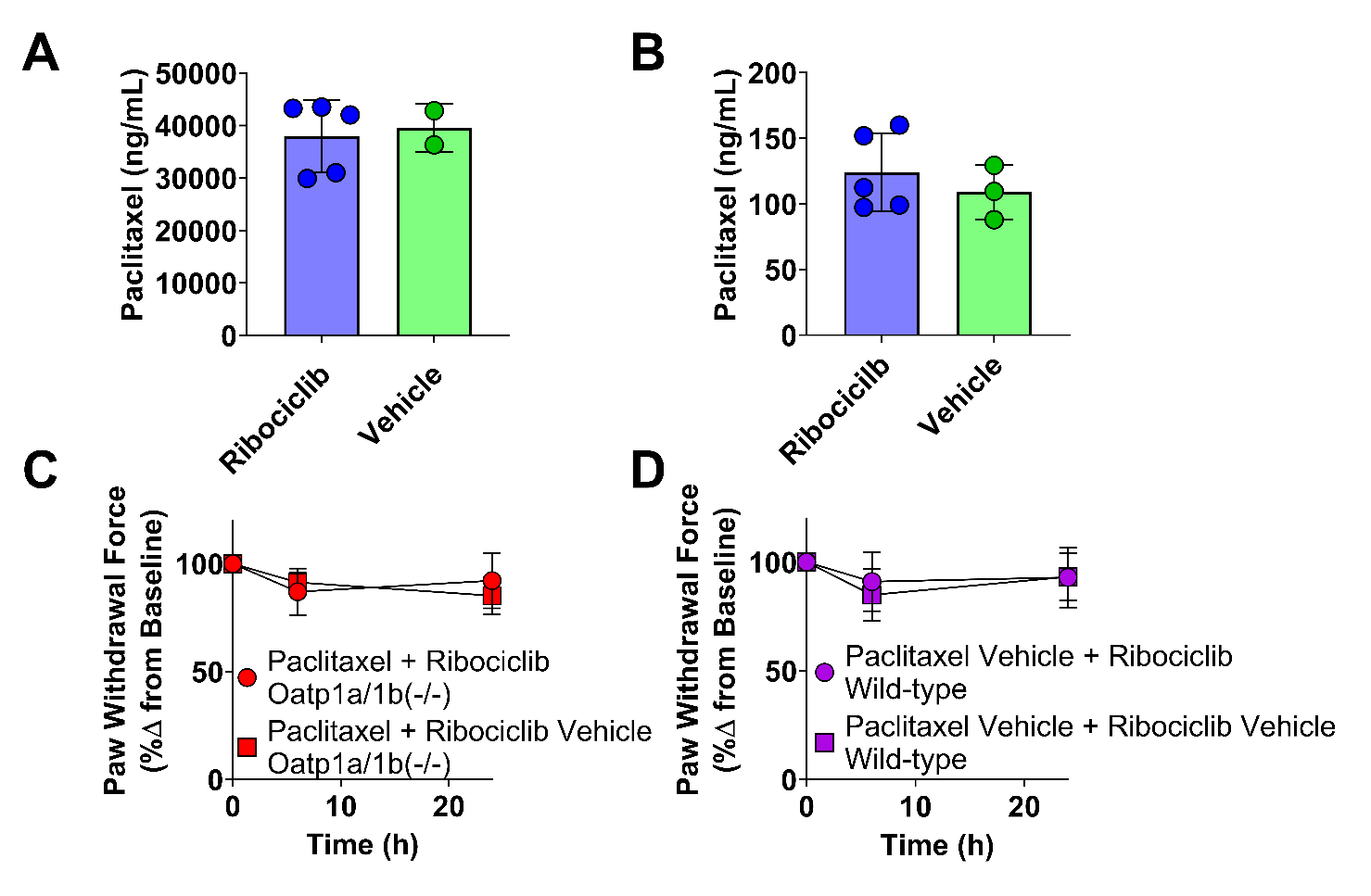


**Supplementary Fig. 7** Pharmacokinetics and pharmacodynamics of paclitaxel in the presence or absence of ribociclib. (**A**) Liver and (**B**) DRG accumulation of paclitaxel when administered in combination with ribociclib. VFH in (**C**) Oatp1a/1b(-/-) mice treated with paclitaxel and ribociclib or vehicle, or (**D**) wild-type mice treated with paclitaxel vehicle and ribociclib or vehicle. n=5 per group, error bars represent SD.

**Supplementary Table 1** Parameters used to calculate R values.

| Drug | Dose (mg) | C_max,ss_ (ng/mL) | PMID | Fu (%) | PMID | Formula Weight | Dose (µM) | C_max_ (µmol) |
| --- | --- | --- | --- | --- | --- | --- | --- | --- |
| Ribociclib | 600 | 3280 | 29059492 | 28 | 30660034 | 434.5 | 1380.90 | 7.55 |
| Palbociclib | 125 | 185 | 26991823 | 15 | 38716900 | 447.533 | 279.31 | 0.41 |
| Abemaciclib | 200 | 298 | 27312735 | 6 | 32799335 | 506.6 | 394.79 | 0.59 |

**Supplementary Table 2** Pharmacokinetic parameters of ribociclib.

| Genotype | N | Sex | Ribociclib Dose  (mg/kg) | C_max_ (µg/mL) | AUC_0-12h_ (µg*h/mL) | AUC_inf_  (µg*h/mL) |
| --- | --- | --- | --- | --- | --- | --- |
| Wild-type | 5 | F | 50 | 4.7 ± 1.2 | 21 ± 11 | 29 ± 13 |
| Wild-type | 5 | F | 100 | 8.0 ± 3.2 | 49 ± 28 | 120 ± 110 |
| Oatp1a/1b(-/-) | 5 | F | 100 | 7.8 ±1.4 | 49 ± 15 | 120 ± 55 |

Data represent the mean ± SD.

**Supplementary Table 3** Pharmacokinetic parameters of triazolam and OH-triazolam.

| Genotype | N | Sex | Analyte | Ribociclib Dose  (mg/kg) | C_max_ (ng/mL) | AUC_0-3.6h_  (ng*h/mL) | AUC_inf_  (ng*h/mL) | C_max_ P:M Ratio | AUC_0-3.6_ P:M Ratio |
| --- | --- | --- | --- | --- | --- | --- | --- | --- | --- |
| Wild-type | 5 | F | Triazolam | 0 | 18 ± 5 | 36 ± 10 | 102 ± 48 |  |  |
| Wild-type | 5 | F |  | 100 | 18 ± 5 | 36 ± 14 | 92 ± 58 |  |  |
| Wild-type | 5 | F | OH-Triazolam | 0 | 36 ± 15 | 67 ± 37 | N/A | 0.51 | 0.54 |
| Wild-type | 5 | F |  | 100 | 25 ± 14 | 44 ± 30 | N/A | 0.72 | 0.82 |

N/A: not applicable. Data represent the mean ± SD.

**Supplementary Table 4** Pharmacokinetic parameters of CDCA-24G.

| Genotype | N | Sex | Ribociclib Dose  (mg/kg) | C_max_ (ng/mL) | AUC_0.25-12h_ (ng*h/mL) |
| --- | --- | --- | --- | --- | --- |
| Wild-type | 5 | F | 100 | 7.6 ± 1.4 | 10.6 ±10.9 |
| Wild-type | 5 | F | 0 | 6.2 ± 1.8 | 21.6 ± 7.1 |
| Oatp1a/1b(-/-) | 5 | F | 0 | 700 ± 50 | 4,400 ± 3,600 |

Data represent the mean ± SD.

| Genotype | N | Sex | Ribociclib Dose  (mg/kg) | C_max_ (ng/mL) | AUC_0-1h_(ng*h/mL) |
| --- | --- | --- | --- | --- | --- |
| Wild-type | 5 | F | 0 | 69 ± 52 | 45 ± 19 |
| Wild-type | 5 | F | 100 | 37 ± 32 | 22 ± 7 |
| Oatp1a/1b(-/-) | 5 | F | 0 | 4669 ± 1480 | 3603 ± 846 |

**Supplementary Table 5** Pharmacokinetic parameters of pravastatin.

Data represent the mean ± SD.

**Supplementary Table 6** Pharmacokinetic parameters of paclitaxel.

| Genotype | N | Sex | Ribociclib Dose  (mg/kg) | C_max_  (µg/mL) | AUC_0-2_ (µg*h/mL) | AUC_inf_ (µg*h/mL) |
| --- | --- | --- | --- | --- | --- | --- |
| Wild-type | 6 | F | 100 | 51 ± 26 | 17 ± 4.8 | 25 ± 7.3 |
| Wild-type | 4 | F | 0 | 65 ± 24 | 18 ± 3.7 | 22 ± 6.2 |
| Oatp1a/1b(-/-) | 4 | F | 0 | 39 ± 15 | 15 ± 6.0 | 19 ± 7.0 |

Data represent the mean ± SD.
